# Incidental Hibernoma With an Atypical Presentation: Diagnostic Approach, Pathologic Correlation, and Lessons Learned

**DOI:** 10.7759/cureus.95600

**Published:** 2025-10-28

**Authors:** Matthew Whalen, Tina Quach, David Greenberg, Emily Reisenbichler, Ramy Shoela

**Affiliations:** 1 Radiology, Saint Louis University School of Medicine, Saint Louis, USA; 2 Orthopaedic Surgery, Saint Louis University School of Medicine, Saint Louis, USA; 3 Pathology, Saint Louis University School of Medicine, Saint Louis, USA; 4 Radiology, Saint Louis University Hospital, Saint Louis, USA

**Keywords:** fdg-avid tumor, hamartoma, hibernoma, lipoma, lipomatous tumor, liposarcoma, pet/ct

## Abstract

Hibernomas are rare fluorodeoxyglucose (FDG)-avid benign tumors originating from brown adipocytes that have characteristics mimicking liposarcomas and lipomas on imaging such as magnetic resonance imaging (MRI) and positron emission tomography/computed tomography (PET/CT). Here, we present a patient who was found to have both an FDG-avid hibernoma and an FDG-avid lung hamartoma, which was initially concerning for metastatic liposarcoma based on imaging. Notable imaging differences that may help differentiate lipomatous tumors include higher maximum standardized uptake values (SUV_max_) on PET/CT for hibernomas compared to liposarcomas and hamartomas. Additionally, on MRI, hibernomas and liposarcomas demonstrate the presence of hypervascularity, heterogeneous composition, and septations as opposed to lipomas. Hamartomas have a heterogeneous composition that may be differentiated by the presence of a cleft-like structure with intranodular fat, low SUV_max_, and calcifications. Understanding the similarities and differences among lipomatous tumors may help guide the physician in devising treatment strategies.

## Introduction

Hibernomas are benign adipocytic tumors that are characterized by their composition of cells that originate from brown adipose tissue, a type of fat that is predominantly present in the fetus and can persist into adulthood [1]. This is in contrast to white adipocytes that are found within typical lipomas. Histological subtypes include the typical, lipoma-like, myxoid, and spindle cell variants, with the typical subtype accounting for ~82% of cases [2]. Hibernomas are often intramuscular and can be found in various locations throughout the body, with the thigh, upper trunk, and neck being commonly affected areas. They typically present as slow-growing masses in young adults [3,4]. These tumors are relatively uncommon, accounting for approximately 1.6% of benign lipomatous tumors. The clinical and radiological features of hibernomas can mimic those of more aggressive lesions like liposarcomas, making biopsy crucial for appropriate diagnosis and management [5]. Hibernomas are typically benign in nature, and recurrence after complete excision is rare. There are no reported cases of malignant transformation [2]. The mainstay of treatment is complete surgical excision of the mass, and patients tend to have a good prognosis after complete excision [6,7]. The goal of the authors is to describe a case in which both an FDG-avid hibernoma and hamartoma were present, which provided diagnostic challenges due to the overlap of imaging characteristics with liposarcomas. We hope the reader learns how to better differentiate hamartomas and lipomatous tumors, including lipoma, liposarcoma, and hibernoma, through the use of imaging and biopsy.

## Case presentation

**Figure 1:**
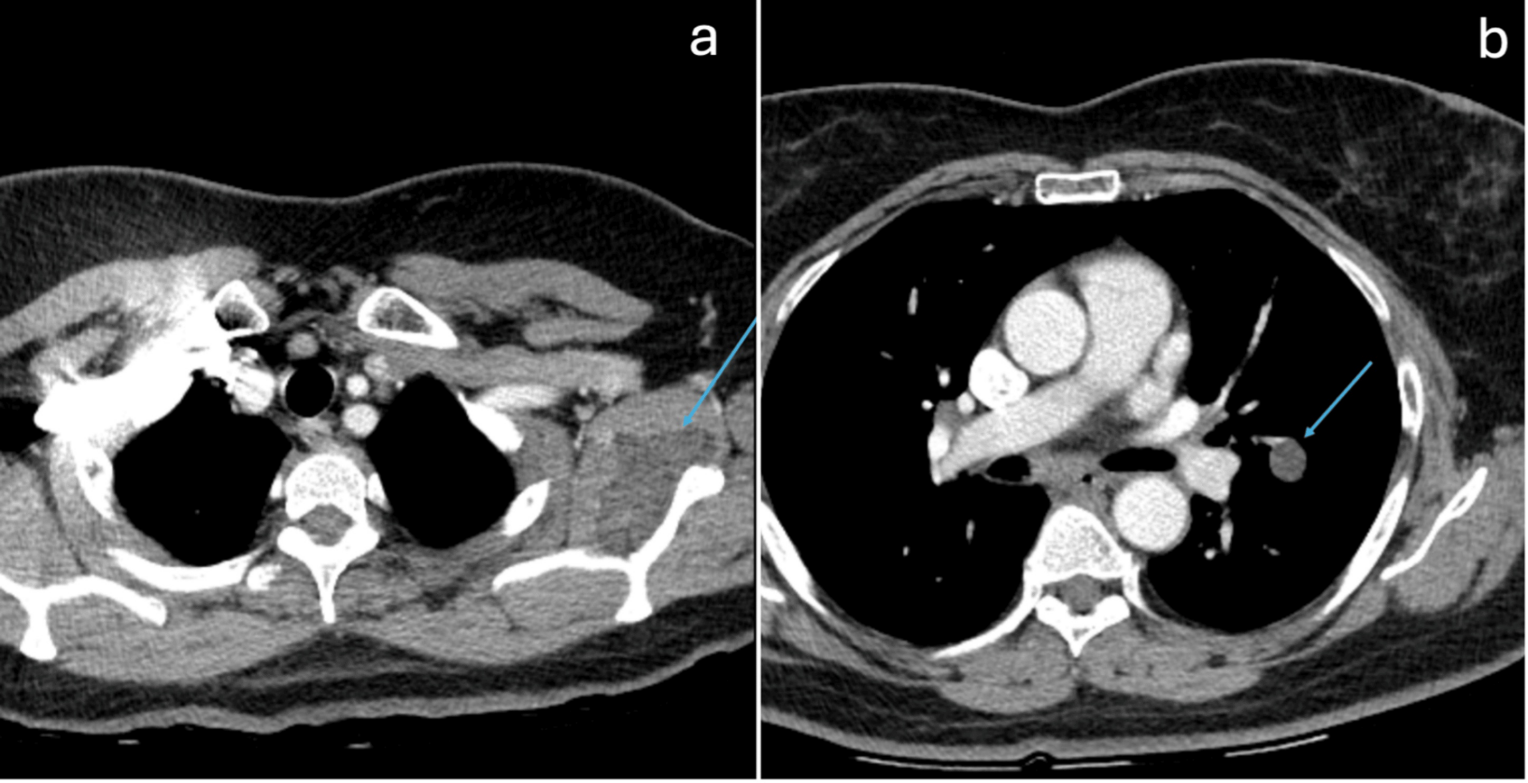
(a) Contrast-enhanced CT of the chest demonstrating a hypoattenuating mass in the left subscapularis muscle that did not measure fat density on CT; (b) Contrast-enhanced CT of the chest demonstrating a hypoattenuating pulmonary nodule. This was also not measuring fat density on CT and was indeterminate and concerning for metastasis at the time.

**Figure 2:**
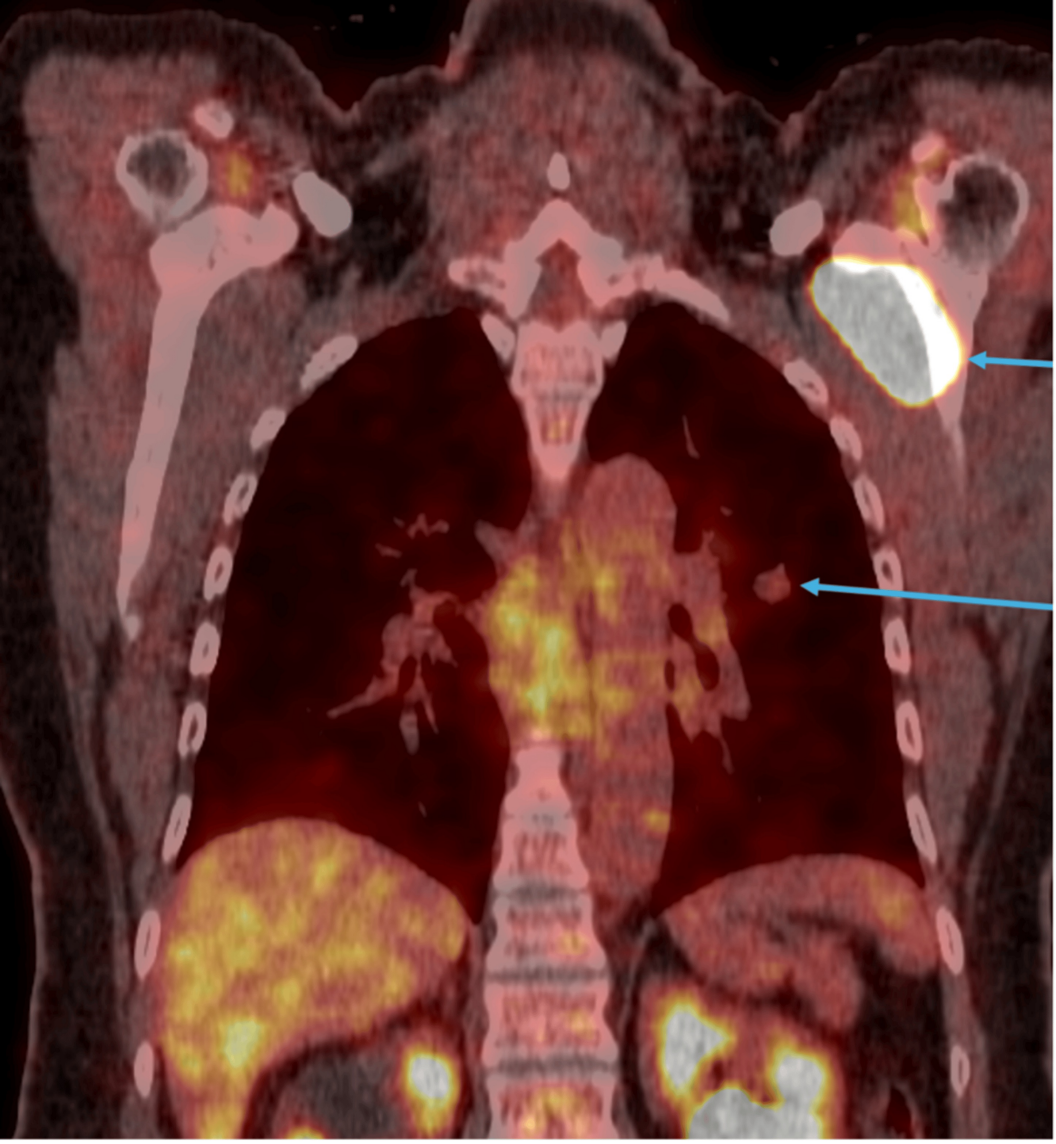
FDG PET/CT imaging showing an intensely avid hypoattenuating mass centered in the left subscapularis measuring up to 6.0 × 2.7 cm axially with an SUVmax of 24.8. Also shown is a 1.7 cm superior lingular nodule that is only mildly qualitatively avid with an SUVmax of 1.8. FDG: fluorodeoxyglucose; PET/CT: positron emission tomography/computed tomography; SUVmax: maximum standardized uptake value

The patient was a 57-year-old woman who initially presented with left shoulder pain for the past two years. The pain was characterized as constant, dull, aching, and radiating to the fingers. She had also had a cough that had been going on for over a month. A chest X-ray was obtained, and a left lung nodule was noted. Further evaluation with a CT scan confirmed the presence of a 1.4 x 1.3 cm cystic lung nodule (Figure 1). The PET/CT scan showed an FDG-avid subscapularis lesion with an SUV_max_ of 24.8. The PET/CT also showed mild avidity in the left lower lobe nodule. The PET/CT did not demonstrate other sites of potential metastatic disease (Figure 2).

**Figure 3:**
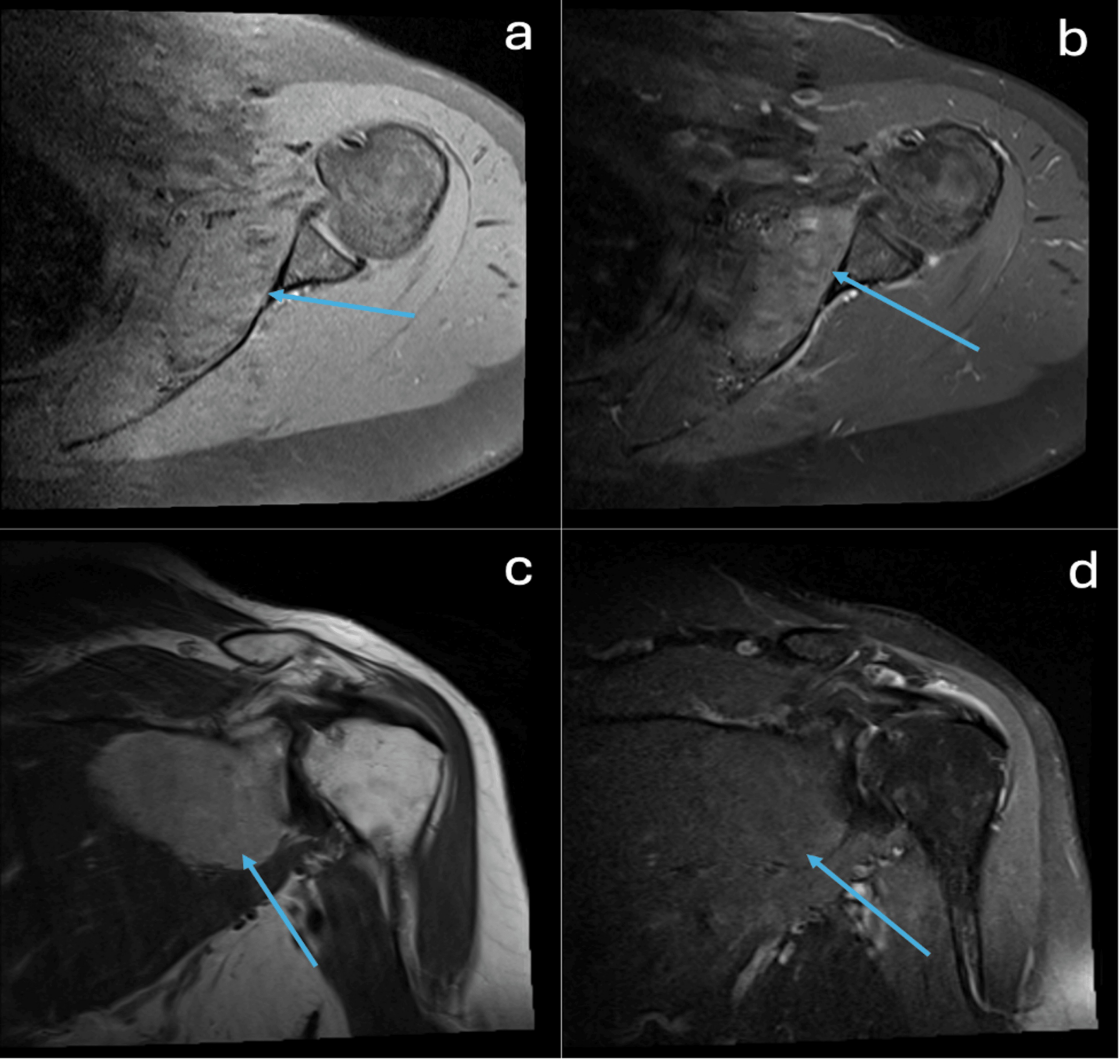
(a) Axial T1-weighted fat-suppressed MRI of the left shoulder pre-gadobutrol demonstrating a lobular 5.8 x 2.4 × 5.4 cm T1 heterogeneous mass. Notably, the mass did not suppress on the fat-suppression images. (b) Axial T1-weighted fat-suppressed MRI of the left shoulder shows patchy enhancement of the mass post-gadobutrol. (c) Coronal T2-weighted MRI of the left shoulder demonstrates that the lesion is hyperintense to skeletal muscle and mildly hypointense to subcutaneous fat. (d) Coronal T2-weighted fat-suppressed MRI of the left shoulder shows incomplete fat suppression of the mass.

**Figure 4:**
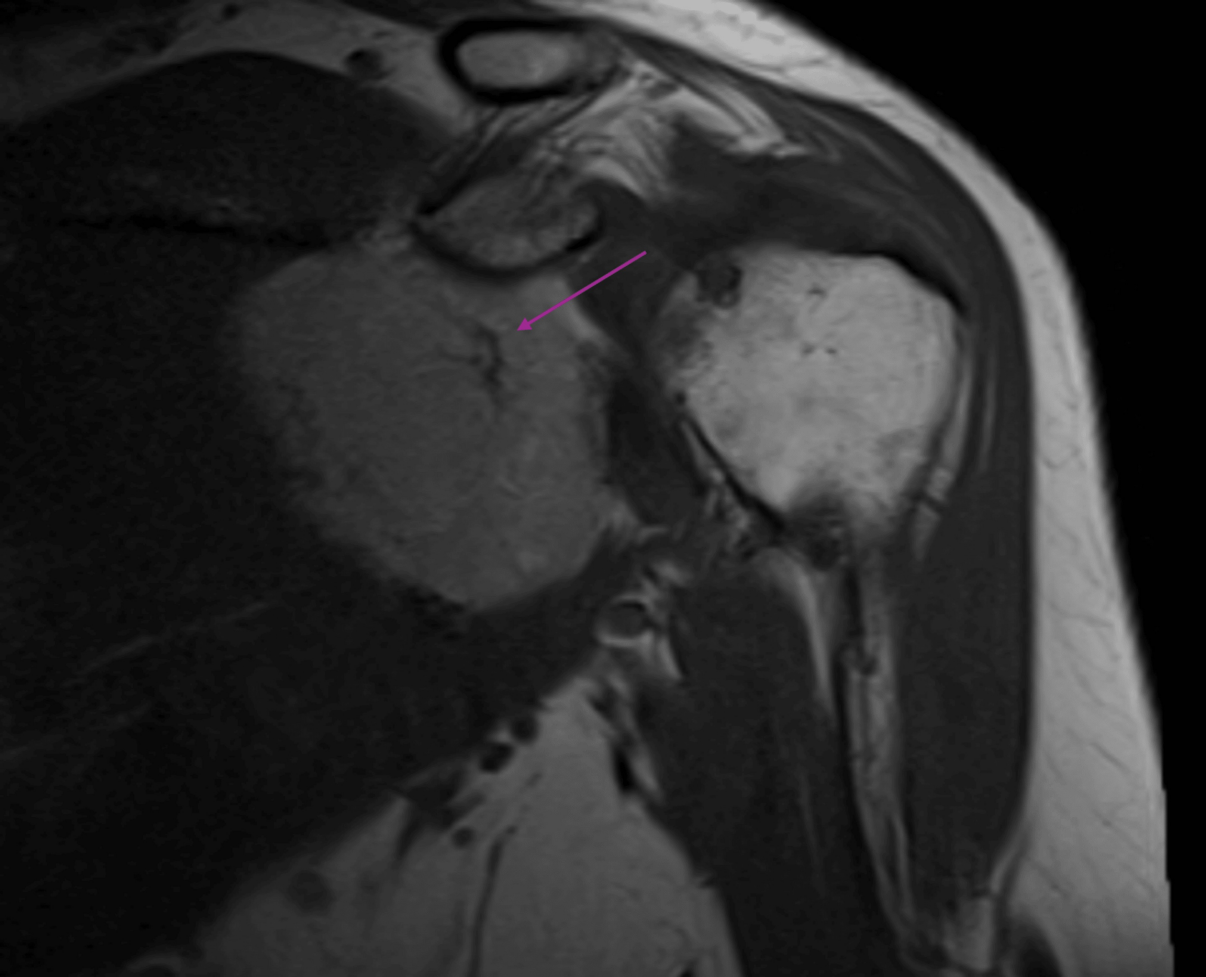
Oblique coronal T1-weighted MRI without fat suppression shows the presence of a mass, which is mildly hypointense to subcutaneous fat, with an intra-tumoral vessel within it (arrow).

**Figure 5:**
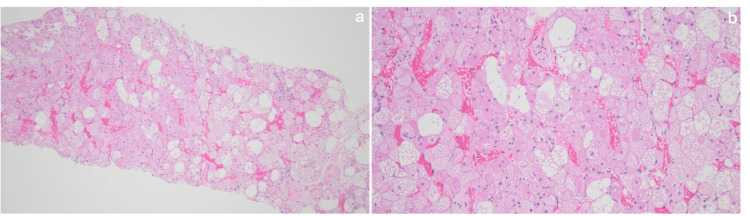
Histological sections of the CT-guided left subscapularis mass biopsy showing multivacuolated adipocytes with granular cytoplasm consistent with brown fat cells; univacuolar adipocytes are also present. Slides were stained with hematoxylin and eosin; image a is at 10x objective power, and image b is at 20x objective power.

Subsequent MRI confirmed a 5.8 x 2.4 x 5.4 cm heterogeneously enhancing mass in the deep aspect of the left subscapular muscle (Figure 3) with an intra-tumoral vessel (Figure 4). The patient was referred to an orthopedic oncologist for further evaluation. Differentials for the shoulder mass at the time included a lipomatous lesion or a soft tissue sarcoma with possible metastasis to the lung. CT-guided core biopsies of both the lung lesion and subscapular soft tissue mass were obtained. Results were consistent with a hibernoma in the left subscapularis (Figure 5) and a chondroid hamartoma in the left lung. In Figure 5, note the increased density of small multivacuolated adipocytes, which is consistent with brown adipose tissue in a hibernoma, compared to the larger mature white adipocytes found in lipomas, which contain a single vacuole. Following biopsy results, the patient elected to undergo excision of the left shoulder mass.

**Figure 6:**
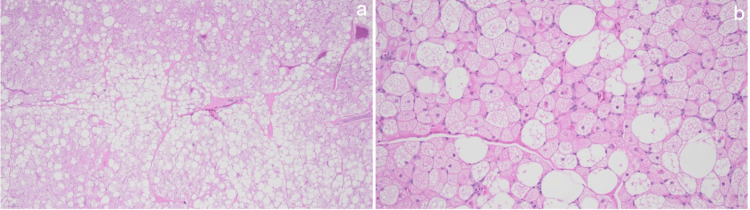
Histological sections of the surgical left subscapularis mass resection showing multivacuolated adipocytes with granular cytoplasm consistent with brown fat cells; univacuolar adipocytes are also present. Slides were stained with hematoxylin and eosin; image a is at 4x objective power, and image b is at 20x objective power.

During removal of the mass, it was noted to extend toward the origin of the subscapularis on the humerus. Due to the location and the challenges associated with reaching the mass, a dual incision was required, including both a posterior scapular incision and an anterior deltopectoral incision. The mass was removed in several lobules. Pathology results and images of the resected hibernoma are shown in Figures 6a-6b, which are consistent with the CT-guided core needle biopsy shown in Figure 5. The patient was evaluated postoperatively at two weeks, six weeks, and six months, with noted gradual improvement in shoulder strength and pain. The patient was referred to pulmonology for further management of the lung lesion, who ultimately elected not to pursue surgical resection since the biopsy results indicated the lesion to be a benign chondroid hamartoma. The patient continues to follow up intermittently with pulmonology for surveillance and lung function tests.

## Discussion

Multiple characteristics of this patient’s presentation are unique from prior documented cases. Current literature on hibernomas is limited, but current studies indicate an increased prevalence in young adults with a mean age of diagnosis at 38 years and a slight predilection for males over females [2,7]. In this case, our patient was a 57-year-old female, outside the typical demographic range. Furthermore, this patient presentation is unusual given the presence of multiple FDG-avid lesions on PET/CT, which would generally increase suspicion for a metastatic process rather than two separate benign pathologies. At the time of obtaining the PET/CT, it was unclear if the FDG-avid lung and subscapular lesions were a singular related disease process or multiple separate processes. The CT-guided biopsies were necessary to discern whether the lesions were related, and the biopsies would ultimately be reported as a lung chondroid hamartoma and subscapular hibernoma, which were separate and unrelated disease processes. This case and further discussion highlight the imaging findings that may help distinguish hibernomas and hamartomas from other FDG-avid and lipomatous lesions.

Hibernomas are reported to be approximately 1.6% of all adipocyte tumors [8-10]; consequently, the appearance of an FDG-avid lesion on PET/CT would put liposarcomas further up on one’s differential over a lipoma or hibernoma. Hibernomas on MRI typically appear as well-defined heterogeneous masses that are hypo- to isointense relative to subcutaneous fat on T1-weighted imaging, mildly hyperintense to fat on T2-weighted and short tau inversion recovery (STIR) sequences with incomplete fat suppression, and variable contrast enhancement. Its internal architecture is generally composed of thin, low-signal septa and prominent tortuous intra-tumoral vessels, unlike lipomas that are generally smooth and without septations [11-14]. Of the MRI findings listed, T2 imaging without full suppression on fat-saturated sequences helps distinguish a hibernoma from a lipoma, and the presence of intra-tumoral vessels helps distinguish it from a liposarcoma (Figure 4) [11-14]. Whereas lipomas can be found either subcutaneously or deep to fascia, hibernomas and liposarcomas are generally deep to fascia [12]. Among lesions found intramuscularly, lipomas are more common than hibernomas. Characteristics of liposarcomas that have increased sensitivity for detection on MRI include deep depth to fascia, thickened septations, enhancing components, and lesion sizes > 10 cm; additionally, less sensitive characteristics include heterogeneous signal intensity and nodules [15]. When comparing the three on PET/CT, lipomas are not found to be FDG-avid, unlike hibernomas and liposarcomas, which helps narrow the differential [16]. The FDG-avidity of hibernomas is due to the increased metabolic activity and presence of glucose transporters, which are characteristics intrinsic to brown adipose tissue. When comparing SUV values on PET/CT between hibernomas and liposarcomas, hibernomas are typically found to be more FDG-avid with an SUV_max_ range of 11.9 - 26.7 versus the range of 4.5 - 8.7 SUV_max_ seen with liposarcomas [17-19]. The presence of septa within the lesion and a heterogeneous composition are characteristics present in both hibernomas and liposarcomas; however, the well-defined margins of a hibernoma can assist in differentiating it from a liposarcoma that has variable margins, depending upon the presence of a pseudocapsule [20]. Table 1 provides a further detailed comparison of lipomas, hibernomas, and liposarcomas to assist with identification [15-27].

**Table 1:** Comparison of image findings and architecture of various lipomatous tumors Citations: [15-27]

Tumor Type	Lipoma	Hibernoma	Liposarcoma
PET/CT FDG-Avidity	No	Yes	Yes
Composition	Homogeneous	Heterogeneous	Heterogeneous
Architecture	Smooth	Nodular Adipose Tissue	Nodular Adipose and Non-Adipose Tissue
Septations	No	Yes, Thin	Yes, Thickened
Margins	Regular, Encapsulated	Regular, Encapsulated	Variable, Pseudo-encapsulated
MRI T1 Signal Intensity	Hyperintense	Mildly Hypointense or Isointense	Myxoid: Hypo- to Isointense, Pleomorphic: Isointense to Mildly Hyperintense
Vascularity	Hypovascular	Hypervascular	Myxoid: Moderate Vascularity, Pleomorphic: Hypervascular
Rim Enhancement	No	No	Yes

Chondroid hamartomas of the lung are also reported to have heterogeneous internal architecture with variable signal intensity due to the mix of mesenchymal tissue within, which may make them appear similar to hibernomas and liposarcomas [28,29]. The architecture of chondroid hamartomas includes enhancing curvilinear lines on MRI that appear as the classically described “cleft-like” structures with intranodular fat. They may also have “popcorn-like” calcifications on CT that help differentiate hamartomas from other lesions such as lipomatous tumors [29]. Chondroid hamartomas are also reported to be much less FDG-avid on PET/CT, with typical SUV_max_ values ranging between 1.0 and 2.5, with malignant lesions often having SUV_max_ greater than 4.4 [30].

## Conclusions

This case highlights an unusual presentation of a hibernoma. This patient’s clinical presentation posed diagnostic challenges, including the presence of a concurrent lung chondroid hamartoma, which broadened the differential diagnosis. Through this case report, we aim to enhance the reader’s understanding of the diagnostic approach and management considerations for hibernomas, hamartomas, and other lipomatous lesions.
